# Seroprevalence of SARS-CoV-2 Infection in the Colombo Municipality Region, Sri Lanka

**DOI:** 10.3389/fpubh.2021.724398

**Published:** 2021-11-12

**Authors:** Chandima Jeewandara, Dinuka Guruge, Inoka Sepali Abyrathna, Saubhagya Danasekara, Banuri Gunasekera, Pradeep Darshana Pushpakumara, Deshan Madhusanka, Deshni Jayathilaka, Thushali Ranasinghe, Gayasha Somathilake, Shyrar Tanussiya, Tibutius Tanesh Jayadas, Heshan Kuruppu, Nimasha Thashmi, Michael Harvie, Ruwan Wijayamuni, Lisa Schimanski, T. K. Tan, Pramila Rijal, Julie Xiao, Graham S. Ogg, Alain Townsend, Gathsaurie Neelika Malavige

**Affiliations:** ^1^Allergy Immunology and Cell Biology Unit, Department of Immunology and Molecular Medicine, University of Sri Jayewardenepura, Nugegoda, Sri Lanka; ^2^Colombo Municipal Council, Colombo, Sri Lanka; ^3^MRC Human Immunology Unit, MRC Weatherall Institute of Molecular Medicine, University of Oxford, Oxford, United Kingdom; ^4^Centre for Translational Immunology, Chinese Academy of Medical Sciences Oxford Institute, University of Oxford, Oxford, United Kingdom

**Keywords:** serosurveillance, SARS-CoV-2, Sri Lanka, Colombo, antibodies, seropositivity

## Abstract

**Background:** As the Municipality Council area in Colombo (CMC) experienced the highest number of cases until the end of January 2021, in Sri Lanka, we carried out a serosurvey prior to initiation of the vaccination program to understand the extent of the SARS-CoV-2 outbreak.

**Methods:** SARS-CoV-2 seropositivity was determined in 2,547 individuals between the ages of 10–86 years, by the Wantai total antibody ELISA. We also compared seroprevalence using the haemagglutination test (HAT) to evaluate its usefulness in carrying out serosurveys.

**Results:** The overall seropositivity rate was 24.46%, while seropositivity by HAT was 18.90%. Although The SARS-CoV-2 infection detection rates by PCR were highest in the population between the ages of 20–60 years of age, there was no statistically significant difference in the seropositivity rates in different age groups. For instance, although the seropositivity rate was highest in the 10–20 age group (34.03%), the PCR positivity rate was 9.80%. Differences in the PCR positivity rates and seropositivity rates were also seen in 60–70-year-olds (8.90 vs. 30.4%) and in individuals >70 years (4.10 vs. 1.20%). The seropositivity rate of the females was 29.70% (290/976), which was significantly higher (*p* < 0.002) than in males 21.2% (333/1,571).

**Conclusions:** A high seroprevalence rate (24.5%) was seen in all age groups in the CMC suggesting that a high level of transmission was seen during this time. The higher PCR positivity rates between the ages of 20–60 are likely to be due to increased testing carried out in the working population. Therefore, the PCR positivity rates, appear to underestimate the true extent of the outbreak and the age groups which were infected.

## Introduction

Eighteen months following the reporting of the first person infected by the SARS-CoV-2 virus, many countries are currently experiencing the third wave with higher caseloads and mortality rates. The steepest increase in the number of cases globally is seen now, as the outbreak is affecting many countries in South and South-East Asia and Latin America (1), which have scarce resources to deal with such large numbers. Similar to the situation in many other South Asian countries, the number of COVID-19 cases is rapidly rising in Sri Lanka. The first patient was detected on the 27th of January 2020, who was a foreign national from China and the first Sri Lankan patient was reported on the 10th of March 2020 (2). Since the detection of the first patient, Sri Lanka went for a strict and extensive lockdown after 10 days (on the 20th of March), which enabled limiting the initial outbreak to certain areas of the Colombo Municipal Council (CMC) area. Although Sri Lanka successfully contained the epidemic until the end of September, with no locally detected cases from August to September 2020, a large outbreak emerged during early October, which rapidly spread island-wide. However, as the CMC is the business capital of the country, and also due to extremely overcrowded living conditions, 32,346/89,817 (36.01%) locally detected cases seen by the end of March 2021, were detected within the Colombo district (3). Of the cases in the Colombo district, 14,416 (44.6%) were identified within the CMC.

**Figure 1:**
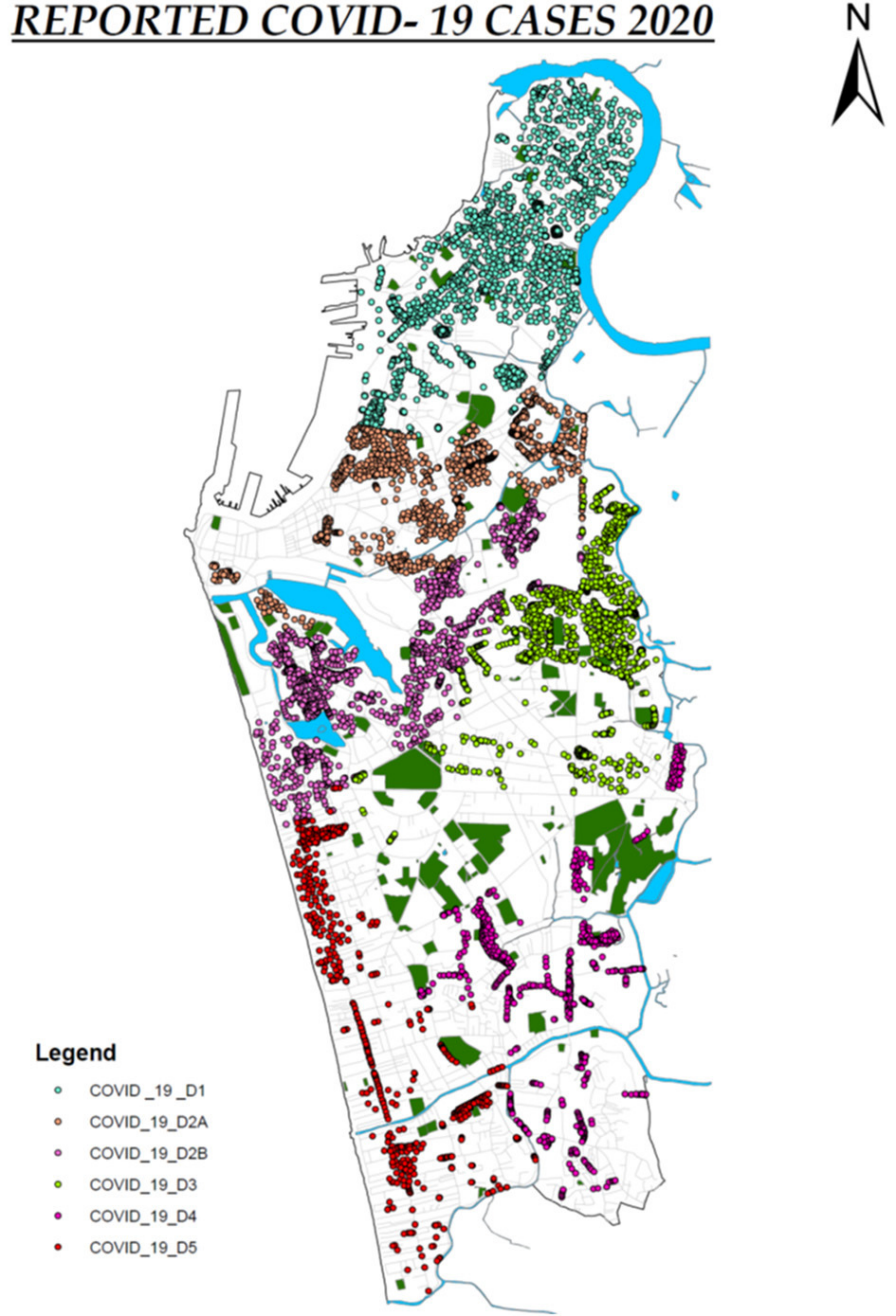
The six districts in the Colombo Municipality Council (CMC) and the locations of cases (identified by qRT-PCR). Each dot denotes an individual person.

The CMC has a population of 561,314 individuals living in an area of 37.3 km^2^. The CMC is divided into six districts: namely D1, D2A, D2B, D3, D4, and D5 (Figure 1). The overall population density in the whole CMC is 20,187.8 individuals/km^2^, although certain areas have a higher density due to poor housing conditions and overcrowding. During the second wave in Sri Lanka, which occurred from October 2020 to March 2021, a rapid rise in the number of cases and intense transmission was seen within this area. However, the number of cases was seen to gradually decline from mid-February to the end of March 2021, with <10 cases per day detected in early April. Administration of the COVID-19 vaccines (Covishield) started on the 29th of January 2021 in Sri Lanka, by initially immunizing health care workers. Immunizing the general public began after the first week of February and due to the high number of cases, the CMC area was prioritized. By mid-March, 20% of the CMC population were vaccinated. Although vaccination itself may have led to a decline in the number of cases in the CMC, it could have also been due to high past infection rates resulting in many individuals being immune to the virus.

In many countries, the reported number of cases do not necessarily reflect the extent of the outbreak, age groups infected and groups at risk, as the majority of infections are asymptomatic and limitations in carrying out quantitative real-time PCR for SARS-CoV2 (qRT-PCR) (4, 5). It has been estimated that surveillance of SARS-CoV2 with qRT-PCR alone may underestimate the true prevalence by tenfold (6). For instance, the overall seroprevalence of COVID-19 in India was found to be 7.1% by end of September 2020, which gives infection rates several folds higher than the actual reported number of cases (7). It is important to carry out serosurveillance studies to understand the true extent of an outbreak in order to understand the future outbreaks that may occur in a particular area and to further understand transmission dynamics and duration of immunity. Therefore, we carried out a serosurveillance study in the CMC at end of January 2021, before the initiation of the COVID-19 vaccination campaign for the general public.

## Methods

### Study Population

2,547 individuals between the ages of 10–86 years were recruited following informed written consent during January 2021 (before administration of COVID-19 vaccines). The population in each of the six districts and the number of individuals from each district recruited for the study are shown in Table 1. During the high transmission rate seen in the CMC from October 2020 to early January 2021, based on the testing strategy adopted in the CMC during that time, which was a test, trace, and isolate; individuals from the different districts of the CMC were subjected to random PCRs. Locations were selected randomly in shopping and housing complexes of the different districts, including outdoor vegetable and fish markets, and office complexes to identify those in the community who were infected with the virus. These particular locations were tested several times during this time period to capture the majority of the working population. As samples for PCR were obtained on only certain days of the week when the team visited the housing complexes and residential areas, the population who underwent PCRs on most days mainly represented the working population. Blood samples were obtained from these participants at the same time when samples were taken from them for these routine random PCR testing for SARS-CoV-2. None of the participants had any symptoms at the time of obtaining blood samples and were not previously diagnosed as been infected with the SARS-CoV-2 virus.

**Table 1:** The number of individuals in each of the CMC districts and the proportion samples from each district.

**District number**	**Total population of the district**	**Number recruited to the study (%)**	**PCR positivity (%)**	**No of PCR+ cases/100,000 population**
D1	131,012	170 (0.13%)	3,663 (2.79%)	2795.92
D2A	137,644	710 (0.52%)	3,290 (2.39%)	2390.22
D2B	61,312	477 (0.78%)	2,676 (4.36%)	4364.56
D3	89,855	466 (0.52%)	2,571 (2.86%)	2861.28
D4	80,839	210 (0.26%)	1,282 (1.59%)	1585.87
D5	60,652	514 (0.85%)	934 (1.54%)	1539.93
Total	561,314	2,547 (0.45%)	14,416	2568.26

Basic demographic details such as age, gender, and prior COVID-19 illness were recorded, and blood samples were obtained to determine the seropositivity status. Ethical approval was obtained from the Ethics Review Committee of the University of Sri Jayewardenepura.

### Detection of Total Antibodies to SARS-CoV-2

SARS-COV-2 specific total antibody (IgM, IgG, and IgA) responses were assessed using Wantai SARS-CoV-2 Ab ELISA (Beijing Wantai Biological Pharmacy Enterprise, China). This assay is specific for the Receptor Binding Domain (RBD) and was shown to have a sensitivity of 98% (8) and was found to be 100% specific based also on control serum samples obtained in 2018, in Sri Lankan individuals. The assay was carried out and results were interpreted according to manufacturers' instructions.

### Haemagglutination Test (HAT) to Detect Antibodies to the Receptor Binding Domain (RBD)

The HAT assay is a very cheap tool, that does not require any specific equipment to detect antibodies as the RBD. In order to compare the usefulness of the HAT assay in comparison to the commercially available Wantai total antibody assay in determining serosurveys, we used the HAT assay in a subset of individuals. The HAT was carried out in a subset of these individuals (*n* = 1,413) as previously described (9) using method (1). The HAT assay detects haemagglutination of red cells labeled with the IH4-RBD reagent. IH4-RBD is a nanobody against a conserved glycophorin A epitope on red cells, linked to the RBD of SARS-CoV-2. Any antibody in the test serum specific for the RBD that can cross-link and agglutinate the red cells will be detected. The HAT was shown to have a sensitivity ~90% and specificity >99% several weeks after a PCR diagnosed symptomatic infection (9). Briefly, red blood cells from an O negative donor diluted in PBS 1:20 (~2% Red Cells) were mixed with 50ul of the IH4-RBD reagent (2ug/ml stock) and 50ul of 1:20 Serum (2.5ul serum in the reaction well) and incubated for 1 h at room temperature to give a dilution of 1:40. Phosphate buffered saline was used as a negative control in place of the diluted serum. At the end of the incubation, the plate was tilted for 20 sec and then photographed. The photograph of the plate was read by two independent readers to examine the “teardrop” formation indicative of a negative result. A complete absence of “teardrop” formation was scored as positive, and any flow of “teardrop” was scored as negative. A HAT titer of 1:40 was considered as positive for the presence of RBD-specific antibodies. We have confirmed that this assay is negative in >99% of individuals prior to infection with SARS-CoV-2 (10).

### Statistical Analysis

Data were analyzed by GraphPad Prism 8 version 8.3. The differences between the Wantai total antibody assay and the HAT in detecting SARS-CoV-2 antibodies were assessed by using the chi-square test. Spearman rank-order correlation coefficient was used to evaluate the relationship between age and seropositivity. All analysis was two-tailed. The Cohen's Kappa values were calculated further with respect to the age groups separately as well as for all age groups combined to assess the agreement between the two raters HAT and WANTAI assays.

## Results

The mean age of the study population was 43.6 years (SD ± 16.07) and out of them, 1,571 (61.7%) were males. The overall seropositivity as measured by the Wantai total antibody assay was 24.46% (623/2,547). The seropositivity rate of the females was 29.7% (290/976), which was significantly higher (*p* < 0.002) than in males 21.2% (333/1,571). The overall seropositivity rate by HAT was 18.9% (267/1,413). Similar to the results obtained from the Wantai assay, the seropositivity rates for females (141/541 = 26.06%) were significantly higher (*p* < 0.00001) when compared to males (126/872 = 14.45%).

**Figure 2:**
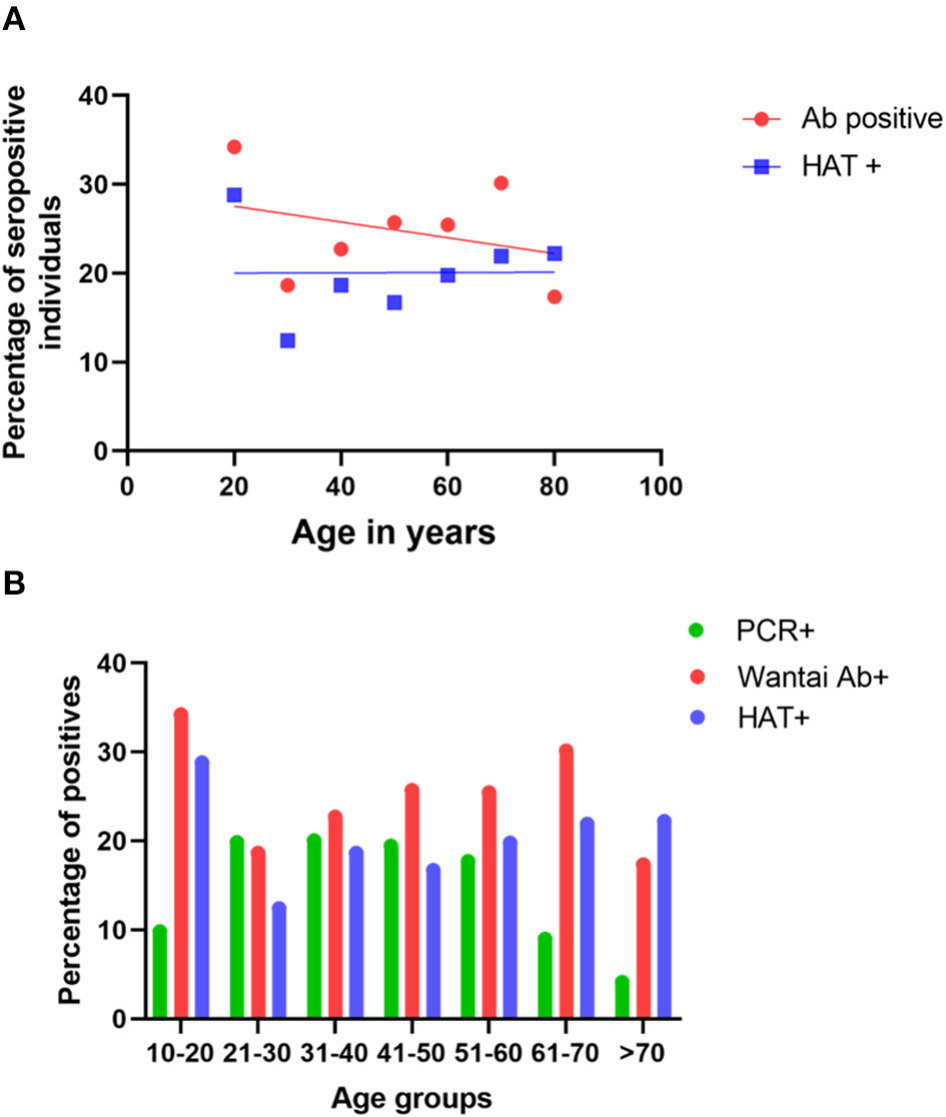
Seropositivity rates and infection rates in different age groups. The seropositivity rates were assessed in each age group by the Wantai total antibody assay (Wantai Ab+) and the HAT assay (HAT+), and the positivity rates of each assay were correlated with the age **(A)**. The PCR positivity rates in each age group and the Wantai (Ab+) and HAT (HAT+) positivity rates were compared in each age group **(B)**.

The age-stratified seroprevalence for the total SARS-CoV2 specific antibodies and the HAT assay is shown in Table 2. There was no significant correlation between the total antibody positivity and HAT positivity (Spearman's *R* = 0.35, *p* = 0.44). There was no significant difference between age and seropositivity rates for either assay (*p* = 0.49, Table 2), with the rates being similar in all age groups (Figure 2A). However, the seropositivity rates were slightly higher in the 10–20 age group based on the results of both the Wantai total antibody assay (34.03%) and HAT (28.57%), compared to other age groups, although this was not statistically significant.

**Table 2:** Age stratified seroprevalence rates for SARS-CoV2 in different age groups with the agreement between HAT and WANTAI.

**Age group**	**Seropositive rates by Wantai *N* = 2,547**	**Seropositive rates by HAT*N* = 1,413**	**PCR positivity rates *N* = 14,416**	**Agreement betweenHAT and Wantai*N* = 1,413Cohen's Kappa(95% CI)**
10–20	65/191 (34.03%)	36/126 (28.57%)	1,418 (9.84%)	0.7407 (0.5721–0.9094)
21–30	83/448 (18.53%)	31/253 (12.25%)	2,864 (19.87%)	0.7109 (0.591–0.8309)
31–40	104/461 (22.56%)	47/253 (18.58%)	2,893 (20.07%)	0.7627 (0.6423–0.883)
41–50	125/484 (25.83%)	45/265 (16.98%)	2,801 (19.43%)	0.6821 (0.5663–0.7979)
51–60	129/507 (25.44%)	54/273 (19.78%)	2,556 (17.73%)	0.7034 (0.5882–0.8186)
61–70	89/293 (30.38%)	39/179 (21.79%)	1,294 (8.98%)	0.6236 (0.4823–0.7648)
Over 70	28/163 (17.18%)	15/64 (23.48%)	590 (4.09%)	1.000 (0.6726–1.161)
Total	623/2,547	267/1,413	14,416	0.718 (0.6673–0.7687)

The Wantai total antibody assay measures the presence of SARS-CoV2 specific IgM, IgG, and IgA antibodies to the RBD, while the HAT assay measures any antibodies to the RBD that can cross-link and agglutinate red cells. The total level of antibody to RBD detected in ELISA is known to correlate with viral neutralizing titer (11), and we have confirmed this for the titer detected in the HAT assay (12). Although a higher number of individuals who were <70 years of age gave a positive result with Wantai assay, in those who were >70 years, the HAT positivity rate was higher (23.48%), although this was not statistically different (*p* = 0.27). In those who were <30 years of age (*n* = 379) and between the ages of 30–60 (*n* = 791), 0.53% (02/379), and 0.76% (06/791), respectively were seropositive by HAT, but negative by Wantai. In the >60-year-old age group (*n* = 243), 1.65% (04/243) who were negative by Wantai were positive by HAT, which was significantly higher (*p* = 0.002) when compared to younger age groups. The HAT assay has been shown to have a higher sensitivity early after infection (9), so these differences may reflect the timing of infection prior to recruitment to the study.

The Wantai and HAT seropositivity rates differed between females and males. While 24.95% (135/541) of females were positive for SARS-CoV-2 by both the Wantai and HAT assays, the positivity rate of males was 13.76% (120/872), which was significantly lower (*p* < 0.002). 1.1% (06/541) of females were only positive by HAT (negative by Wantai), whereas 0.69% (06/872) of men were only positive by HAT.

### qPCR Positivity in Different Age Groups vs. Seropositivity

As many cases were detected in the CMC during this period, qRT-PCRs were carried out on all primary contacts and most of the secondary contacts and also randomly to identify infected individuals in the community. Of the total PCR positive individuals (*n* = 14,416), that included symptomatic individuals, asymptomatic primary and secondary contacts, and those identified by random screening, 11,108 (77.1%) were between the ages of 20–60 years. While the PCR positivity rates (infection detection rates) were between 17.7 to 20.05% in the age groups from 20 to 60 years, it was 9.8% in 10–20-year-old individuals and 8.9% in 60–70-year-olds (Figure 2B) possibly because of increased random qRT-PCR testing carried out in the working-age groups, leading to increased detection of infection. Although infection detection rates were less in those <20 years and those >60 years, possibly due to the lesser number of qRT-PCR tests carried out, the seropositivity rates were higher in the 10–20-year-old age group (Figure 2B).

## Discussion

In this study, we have carried out a serosurveillance for COVID-19 in the CMC area, which experienced the highest number of cases (16.1%) in the whole country. Our data showed that the overall seropositivity rate was 24.46% in this area, until the end of January 2021. We also compared the HAT to determine the suitability of this test in serosurveillance studies, as this is a cheap, easy-to-use test, which requires no equipment and therefore, would be suitable for lower-income countries. Although the overall seroprevalence rates by the HAT were slightly lower (18.9%) than the seropositivity rates by the Wantai total antibody assay, it appeared to give satisfactory results in all age groups, although the sensitivity was lower. Furthermore, the HAT titres were shown to correlate with the live virus neutralizing antibody assays (12). Therefore, it is possible that the HAT assay gave a negative result in those who do not have neutralizing antibodies.

During the period of the study, the testing strategy adopted by the CMC was to test, trace and isolate, in order to identify all possible infected individuals in the community. This was to then isolate them to prevent the transmission of infection. Therefore, qRT-PCRs were carried out in many shopping and housing complexes, residential areas, office complexes, and markets, and the individuals were encouraged to get tested in order to identify the infected. Although the testing strategy was targeted to identify all possible infected individuals in the community, the prevalence of SARS-CoV-2 infection assessed by the seroprevalence studies was several-fold higher than the prevalence of COVID-19 based on PCR positivity, which is 2,568/100,000 population. Based on the seropositivity rates of 24.46%, 138,276 individuals are likely to have been infected with the SARS-CoV-2 virus compared to the reported PCR positive cases of 14,416. Therefore, infection detection rates by PCR appeared to have underestimated the actual number of infections by 9.59-fold, which is not surprising as the random PCRs were mainly carried out in the working population and less frequently in those who were confined to their houses. Furthermore, neither of these assays (PCRs or antibody assays) were done by random sampling, but on those who volunteered for these tests. Therefore, a direct comparison of infection rates detected by PCR and seropositivity rates is inappropriate. However, such differences have been reported elsewhere and are comparable to the results of other studies. Serologically detected cases have been shown to outnumber the virologically confirmed SARS-CoV-2 infection by 10-fold possibly due to these differences in many countries (6). In this study, we evaluated the usefulness of the HAT to determine seroprevalence, which showed a seropositivity rate of 18.9%. Although the seropositivity rates from HAT were slightly lower than from the Wantai total antibody assay, our data show that the HAT assay appears to be a sensitive tool, that can be used to carry out serosurveys in resource-poor settings as it is a cheap assay that does not require any equipment.

Although the overall seroprevalence was 24.46%, certain districts in the CMC (D2A, D2B, and D3) had higher seroprevalence rates (26.2–39%) compared to D4 which only had a seroprevalence rate of 3.33%. These overall differences between the districts reflect the population density and the housing conditions in these districts, with the districts with high seroprevalence having more overcrowded areas, with poor housing conditions. The differences in the seroprevalence rates in different districts could also be due to differences in the control measured adopted. For instance, in D1, although the seroprevalence was 14.76%, certain areas in this district had a very high infection rate as determined by PCR positivity. Due to early detection of SARS-CoV-2 infection in certain areas in this district, these areas were isolated very early, and therefore, it would have curtailed the spread to the rest of the D1 district resulting in fewer infections. Such similar differences have been observed in many states in India, where the slum areas reported seroprevalence rates between 52.6 to 58.7% compared to 12–17.9% in non-slum areas (13). Although the overall seroprevalence rates in the CMC was less than urban areas in India, it was higher than many areas in Europe (Spain, Sweden, Switzerland, and Germany), which reported a seroprevalence between 5 to 13.6% and Iran (22.16%), which reported higher infection rates (14–17). However, the use of different antibody assays, which have a varying degree of sensitivity and specificities in these different studies could result in such differences.

These seroprevalence studies in other countries were carried out during 2020, when Sri Lanka did not experience any large-scale outbreaks of COVID-19, as the first large outbreak only occurred in October 2020. In fact, a serosurvey carried out in May 2020, in an area in the CMC where the first outbreak was seen in April–May 2020, showed a seroprevalence rate of 1.5% in individuals living in that area (PLoS ONE accepted). Therefore, the large outbreak that was seen mainly in the CMC area, from October 2020 to the end of January 2021, appeared to have rapidly spread, resulting in 24.46% of individuals being infected within 4 months.

Although the SARS-CoV-2 infection detection rates were highest in the population between the ages of 20–60 years of age (working population, because of increased testing), the seropositivity rates were equal among all age groups. For instance, although the PCR positivity rates were 9.8% in 10–20-year-olds, 8.9% in 60–70-year-olds, and 4.1% in individuals >70 years, their seropositivity rates were 36.8, 32.1, and 26.6%, respectively. Therefore, it is likely that individuals of all age groups were equally infected by the SARS-CoV-2 virus, although the infections were only detected by PCR in working age groups, due to the increased number of tests that were done in the working population. Interestingly, the seroprevalence rates were significantly higher in females (29.7%) when compared to males (21.1%). Similar results were obtained with the HAT assay with seropositivity rates being significantly more in females (24.9%) compared to males (13.76%). Although the reasons for these differences are not clear, it is possible that more females were exposed to other infected individuals, while carrying out their daily activities using common water and washroom facilities, available in these overcrowded housing settings.

In summary, we assessed the seroprevalence in the CMC area in Colombo, which experienced the highest number of cases from October 2020 to January 2021. Our data show that the serologically detected infections outnumbered the PCR detected cases by almost 10-fold, which demonstrates the importance of seroprevalence studies in identifying the true extent of an outbreak.

## Data Availability Statement

The original contributions presented in the study are included in the article/supplementary material, further inquiries can be directed to the corresponding author/s.

## Ethics Statement

The studies involving human participants were reviewed and approved by Ethics Review Committee, University of Sri Jayewardenepura, Sri Lanka. Written informed consent to participate in this study was provided by the participants' legal guardian/next of kin.

## Author Contributions

CJ, GM, and AT: study design. CJ and DG: project administration. DG, CJ, HK, and RW: recruitment of study participants. IA, SD, BG, PP, DM, DJ, TR, ST, TJ, NT, and MH: carrying out experiments. LS, TT, PR, and JX: laboratory assay development. GS and GM: data analysis. GM: writing the manuscript. CJ, GO, and AT: editing and proofreading the manuscript. CJ, GO, AT, and GM: funding. All authors contributed to the article and approved the submitted version.

## Funding

TT was funded by the Townsend-Jeantet Charitable Trust (charity number 1011770) and the EPA Cephalosporin Early Career Researcher Fund. AT was funded by the Chinese Academy of Medical Sciences (CAMS) Innovation Fund for Medical Science (CIFMS), China (grant no. 2018-I2M-2-002).

## Conflict of Interest

The authors declare that the research was conducted in the absence of any commercial or financial relationships that could be construed as a potential conflict of interest.

## Publisher's Note

All claims expressed in this article are solely those of the authors and do not necessarily represent those of their affiliated organizations, or those of the publisher, the editors and the reviewers. Any product that may be evaluated in this article, or claim that may be made by its manufacturer, is not guaranteed or endorsed by the publisher.
